# Core indicators evaluation of effectiveness of HIV-AIDS preventive-control programmes carried out by nongovernmental organizations. A mixed method study

**DOI:** 10.1186/1472-6963-11-176

**Published:** 2011-07-28

**Authors:** Anna Berenguera, Enriqueta Pujol-Ribera, Concepció Violan, Amparo Romaguera, Rosa Mansilla, Albert Giménez, Carlos Ascaso, Jesús Almeda

**Affiliations:** 1 Research Department, Primary Health Care Research Institute (IDIAP-Jordi Gol), Catalan Health Institute (ICS), (Gran Via de les Corts Catalanes 587 àtic), Barcelona, (08007), Spain; 2 Primary Health Department Costa de Ponent, Catalan Health Institute (ICS), IDIAP-Jordi Gol, Gerència Territorial Metropolitana Sud, Hospital Duran i Reynals,(Gran Via, km 2,7 s/n 1ª planta), L'Hospitalet del Llobregat, (08907), Spain; 3 AIDS Programme. Public Health Department. Ministry of Health. Government of Catalonia, (Roc Boronat, 81-95), Barcelona, (08005), Spain; 4 CIBER, Epidemiology and Public Health (CIBERESP), Instituto de Salud Carlos III, (Melchor Fernández Almagro, 3-5), Madrid, (28029), Spain; 5 Public Health Department, Medicine Faculty, Barcelona University, (Casanova 143), Barcelona, (08036), Spain

## Abstract

**Background:**

The number of nongovernmental organizations working on AIDS has grown. There is great diversity in the type of activities and population groups that have been targeted. The purposes of this study are: to describe and analyze the objectives and HIV-AIDS preventive activities that are carried out by the AIDS-NGOs that work with AIDS in Catalonia and that receive subsidies from the Department of Health; and to develop a comprehensive proposal for measurable and agreed upon core quality evaluation indicators to monitor and assess those objectives and activities that can have an impact on the fight against inequalities and stigmatization, and incorporate the perspectives of the service providers and users.

**Methods:**

A mixed method study has been carried out with professionals from the 36 NGOs that work with HIV/AIDS in Catalonia, as well as their users. This study achieved the completeness model using the following phases:

1. A systematic review of AIDS-NGOs annual reports and preparation of a catalogue of activities grouped by objectives, level of prevention and AIDS-NGOs target population; 2. A transversal study through an ad-hoc questionnaire administered to the AIDS-NGOs representatives; 3. A qualitative study with a phenomenological approach through focus groups, individual interviews and observations; 4. Consensus meetings between AIDS-NGOs professionals and the research team using Haddon matrices in order to establish a proposal of evaluation indicators.

**Results:**

The information was classified according to level of prevention and level of intervention. A total of 248 objectives and 258 prevention activities were identified. 1564 evaluation indicators, addressed to 7 target population groups, were produced. Thirty core activities were selected. The evaluation indicators proposed for these activities were: 76 indicators for 15 primary prevention activities, 43 for 5 secondary prevention activities and 68 for 10 tertiary prevention activities.

**Conclusions:**

The results could help to homogeneously assess the preventive-control activities carried out of AIDS-NGOs. The proposed indicators could help the professionals to improve the evaluation of the preventive-control AIDS-NGOs activities. Furthermore, the Haddon matrix enables us to identify deficiencies of activities at intervention levels and strategies to bear in mind in order to enhance the future AIDS prevention programs.

## Background

The 2001 Declaration of Commitment on HIV/AIDS and the 2006 Political Declaration on HIV/AIDS adopted by the United Nations General Assembly are the guiding forces of the global response to AIDS. The process of preparing country progress reports should involve all partners in AIDS response and should provide an opportunity for reflection on the national response, its achievements as well as its shortcomings, in order to reach universal goals. Monitoring the response to the AIDS epidemic is essential to ensure that investments in AIDS achieve the expected results in health and society [1].

HIV-related Non Governmental Organizations (AIDS-NGOs) work to promote prevention and to provide care and help to people affected by HIV in most countries of the world. The role of these AIDS-NGOs has been critical in the fight against HIV infection since the onset of the epidemic [2]. The activities of the AIDS-NGOs are important both around the world and in Catalonia (one of 17 Autonomous Communities in the North-East of Spain with 7,364,078 inhabitants in 2008) [3]. These activities are complementary and are often leaders of public and private health systems in various countries. AIDS-NGOs have lead the initiative against HIV and act as a bridge and "communications space" between the most vulnerable sectors of the population (because of socioeconomic inequalities) and health services [4]. AIDS-NGOs are the largest providers of preventive activities against HIV/AIDS, particularly amongst high-risk behavior groups: commercial sex workers, injecting drug users, men who have sex with men, youths in high-risk situations, prisoners and immigrants and persons living with HIV/AIDS [5,6]. Furthermore, they have wide experience of working at the community level, and their autonomous nature allows them to respond more quickly and employ innovative methods [7].

Since the inception of the AIDS National Program in Catalonia, the promotion and coordination of activities carried out by AIDS-NGOs has been emphasized. To support the AIDS-NGOs, since 1992 the Department of Health has provided annual subsidies that have progressively increased to reach 1.4 million Euros in 2007 [8-10]. There has been an increase in the prevention, health promotion and care activities of AIDS-NGOs [6,11].

The number of AIDS-NGOs and their activities has grown to meet the demand and the needs of individuals in preventive materials, determination of serostatus and HIV care. For this reason there is great diversity in the type of activities, as well as in the social levels and population groups that have been targeted.

In Spain, Peiró et al. systematically classified and evaluated the activities of national AIDS programs, including AIDS-NGOs activities using the Haddon matrix. This study concluded that there is much heterogeneity in the structure and indicators of the various programs. Concepts such as goals, objectives and priorities are poorly defined. Moreover, in all countries the implementation of preventive programs shows a predominance of individual interventions over social ones [12].

A more recent report, the Health Plan of the Community of Valencia 2005-2009 [13] includes a description of objectives and lines of action for HIV prevention based on the Haddon matrix axes [14-16]. Other autonomous communities in Spain such as the Canary Islands and Asturias have also provided a basis for developing evaluation indicators for all activities [17,18]. In Spain, the "AIDS National Programme" of the Ministry of Health addresses the development of preventive programs for HIV and other sexually transmitted infections in individuals that work as commercial sex workers. The plan suggests both qualitative and quantitative evaluation indicators for these activities [9].

Moreover, the methodology that has been proposed to compare the different ways to prioritize issues and to forecast a policy's application in terms of results and outcomes is the Haddon Matrix. This matrix is a way to synthesizes the enormous amount of information available in strategy documents, shorten the time needed to produce an assessment, and improve a policy's value by comparing it with external models. In this paper we use this theoretical framework developed with the explicit intention of covering the need of the AIDS National Program of Catalonia to know what preventive and control interventions are done by AIDS-NGOs with the objective of improving the praxis of these activities and establishing new strategies for the AIDS National program [12]. Furthermore, a proposal of measurable indicators to monitor and assess each activity done by these organizations will be developed.

It is important to provide key constituents who are actively involved in a country's response to AIDS with essential information on core indicators that measure the effectiveness of the national response. The indicators will help to ensure the consistency and transparency of the process used by national governments. Increasingly, countries are strongly encouraged to integrate the core indicators into their ongoing monitoring and evaluation activities [19].

The key motivation for undertaking this work is to propose quality indicators which include users' satisfaction, psychosocial aspects that can have an impact on the fight against inequalities and stigmatization, and the perspectives of professional service providers. Until now, in many evaluation proposals, these aspects were not really addressed in the HIV literature, while their consideration would permit an important step toward the incorporation of strategies for evaluation of NGO prevention and more comprehensive control activities.

The purposes of this study are: to describe and analyze the objectives of and the HIV-AIDS preventive activities carried out by the AIDS-NGOs that work with AIDS in Catalonia and that receive subsidies from the Department of Health; and to develop a more comprehensive set of measurable and agreed upon core quality evaluation indicators to monitor and assess those objectives and activities that can have an impact on health and the fight against inequalities and stigmatization, and incorporate the perspectives of service providers and users.

## Methods

**Figure 1:**
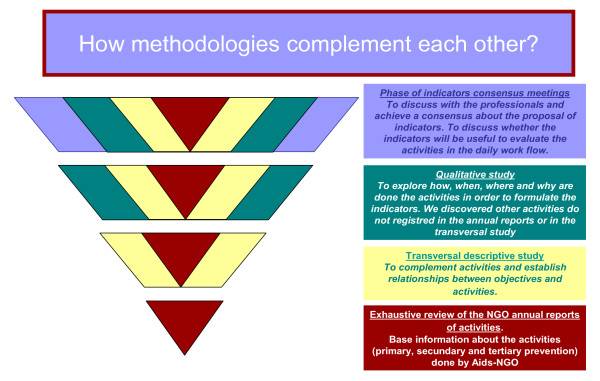
**Methodological Strategy**.

A mixed method strategy was used in order to achieve the objectives of the study. The way to combine quantitative and qualitative research was by completeness, in order to achieve more complete and comprehensive answers to research questions. The strategy was divided into four phases and there was full data integration of each phase (each new phase includes the findings of previous phases) [20-23] (Figure 1).

### Phase 1-Systematic and exhaustive review of the AIDS-NGOs annual reports

A systematic reading and review of the 36 annual reports from the all AIDS-NGOs working in Catalonia and which received subsidies from the Catalan Government in 2006. The annual reports of AIDS-NGO were provided by the AIDS National Program exclusively for this study.

From this systematic reading, we found objectives and activities of prevention were classified according to the following criteria [12,15,16]: AIDS-NGOs target population (commercial sex workers, injecting drug users, men who have sex with men, youths in high risk situations and persons living with HIV/AIDS), levels of prevention (primary, secondary and tertiary), and levels of intervention (individuals, the individual's immediate environment, the external environment and the social environment). This classification was the first step in constructing Haddon matrices. The Haddon matrix offers a conceptual framework that facilitates the analysis of health problems, taking into consideration a three dimensional grid: on the x-axis, the time dimension: primary prevention, secondary prevention and tertiary prevention; on the y-axis, the setting of the intervention: individual factors, immediate environment, external environment and a fourth level that includes the social system and the current regulations. The third axis or dimension includes actions designed to decrease gender, age, ethnic, socio-economic and cultural inequalities [12,15,16].

From this synthesis, a database relating the objectives and activities described in the AIDS-NGOs annual reports was created.

What new information does this methodological phase of the study provide?

This phase provides new basic information on the objectives of and the activities carried out by the AIDS-NGOs as described in the annual reports these organisations prepare for the Health Department. A Haddon matrix has also been constructed with the activities classified by strategy, level of prevention, and level of intervention.

### Phase 2-Transversal study

An ad-hoc questionnaire was constructed in order to identify activities and objectives classified according to AIDS-NGOs target populations that were not explicit in the AIDS-NGOs annual reports.

A pilot study was carried out in 2008, using a cognitive interview which allowed assessment of the understanding and applicability of the questionnaire [24,25].

The questionnaire was self-administered and it was sent by mail to all the 36 AIDS-NGOs. It consisted of 15 questions, (5 open questions, 7 closed questions and 3 multiple choice questions with non-mutually exclusive answers) and included the followed sections: (1) Profile of the individual responding to the questionnaire, (2) Information regarding the AIDS-NGOs target population, (3) Objectives of the AIDS-NGOs and preventive activities of the program, (4) Preventive activities carried out by the AIDS-NGOs, (5) Agreement to participate in the future consensus meetings between AIDS-NGOs professionals and the research team to establish an evaluation indicators proposal. In each section, there was one open question to express opinions and experiences that were not covered by the questionnaire.

To facilitate the highest percentage of responses, two rounds of confirmatory phone calls were conducted and a descriptive analysis (absolute frequencies and percentages) was performed.

What new information does this methodological phase of the study provide?

The results of the transverse study complement those of the first phase of the elaboration of the Haddon matrix based on the systematic study of the reports. In this methodological phase we have also been able to establish a relationship between objectives and activities.

### Phase 3-Qualitative study of phenomenological perspective

A qualitative study was conducted in order to identify the experiences that professionals in the Catalan AIDS-NGOs had in preventive activities, the potential areas of improvement of the activities, the professionals' evaluation of their activities, the experiences and practices of AIDS-NGOs with regard to HIV infection and prevention, and elements that enhanced their relationship with the AIDS-NGOs.

A theoretical sampling (professional) and opportunistic sample (participants) was proposed. Professionals and users from 36 AIDS-NGO funded by the Health Department to take part in the study. For the professionals, a theoretical sampling based on the previous definition of the characteristics of the participants has been carried out to obtain the greatest variety and discursive wealth to reach data saturation. Variables used to define the informant profile for the professionals of AIDS-NGO are: AIDS-NGO target population (commercial sex workers, injecting drug users, men who have sex with men, youths in high-risk situations, prisoners and immigrants and persons living with HIV/AIDS), age, sex, professional profile, setting (urban or rural) and years of experience.

Due to the difficulties of the theoretical sampling, an opportunistic sampling was finally chosen for the AIDS-NGO users. However, heterogeneity criteria were taken into account. Variables used to define the users are: AIDS-NGO target population, age, sex, nationality, serostatus, and time in contact with the NGO [26].

Different techniques have been used [27]. For the professionals, focus group [28] and triangular groups [29]. In the focus groups, interaction is the instrument to stimulate the individual speech [30]. For the users, semi-structured interviews (because during the interviews some sensitive issues may arise) [30] and open, focused and non-systematic observation of theatre performances in teenagers and young adults have been carried out [31].

Data collection was between February and June 2008. Focus groups took place in a neutral place (IDIAP Jordi Gol) and they included a moderator and an observer. The semi-structured interviews took place in the working place of the users or at the AIDS-NGO places. The observations were made in two secondary schools.

A thematic interpretive content analysis was conducted by three analysts. More details about the methodology of this phase have been published elsewhere [32].

What new information does this methodological phase of the study provide?

This methodological phase of the study has permitted a deeper knowledge of how AIDS-NGO professionals carry out their activities. It has also allowed exploration of the barriers professionals encounter in carrying out evaluation. Users also took part in this methodological phase of the study, in which their knowledge and perception of HIV-AIDS risks was explored.

### Phase 4-Consensus meetings between NGO professionals and the research team

From the synthesis of the information obtained in phases 1, 2 and 3, the Haddon matrices described in phase 1 were completed. Later, consensus meetings were held between five AIDS-NGOs professionals and four members of the research team, in order to review and redevelop the Haddon matrices and to identify new preventive activities when possible.

The AIDS-NGOs professional selection was carried out considering the inclusion of different professional profiles, work with different AIDS-NGOs target groups, years of antiquity and the geographical setting.

From the Haddon matrices, a proposal of qualitative and quantitative evaluation indicators was developed, both for process and outcomes of activities carried out by AIDS-NGOs. The structure and methodology that was followed in order to develop the indicators proposal is shown in tables 1 and 2 [33].

**Table 1:** Structure of the proposal of indicators

AIDS-NGOs target population			
**Prevention and/or intervention level**			

**ACTIVITY**			

**Objective**			

**PROCESS MEASURE**	**Indicator description**	**Source of Data**	**Data Collection Frequency**

Measures the process carried out, directly or indirectly, on the participant. A good process indicator is one that is based on activities closely related with the outcome of the performance. These indicators reflect the standards or criteria of adequacy according to the professionals' consensus and from the literature.	Format of the indicator	Institution, person or document from which data are obtained for elaborating the indicators	It measures the frequency in which this process indicator should be evaluated

**OUTCOME MEASURE**	**Indicator description**	**Source of Data**	**Data Collection Frequency**

Measures the effect of the process on the participant. The grade in which this outcome is influenced by other independent process	Format of the indicator	Institution, person or document from which data are obtained for elaborating the indicators	It measures the frequency with which this process indicator should be evaluated

**Table 2:** Criteria used to construct and to evaluate the indicators

Criteria used to construct the indicators
1. The indicator measured performance of an intervention or treatment with potential health benefits for the patient
2. The indicator was supported by scientific evidence or professional consensus
3. The indicator was under the control or influence of the care provider or health plan
4. Evidence to evaluate the indicator could be found in the medical record and its absence from the record could be considered a marker for poor quality

**Criteria used to evaluate the indicators**

***Criteria for validity***

1. Adequate scientific evidence of professional consensus exists supporting the indicator
2. Identifiable health benefits to patients who receive care specified by the indicator
3. Health professionals with significantly higher rates of adherence to an indicator would be considered higher quality providers
4. Most factors that determine adherence to an indicator are under the control of the health professional (or are subject to influence by the health professional).

***Criteria for feasibility***

1. The information necessary to determine adherence is likely to be found in a typical medical record
2. Estimates of adherence to the indicator - based on medical record data - are likely to be reliable and unbiased.
3. Failure to document the indicator is itself a marker for poor quality

Notes: Table adapted from Monitoring the Declaration of Commitment of HIV/AIDS: Guidelines on construction of core indicators: 2010 reporting.

Finally, core indicators were selected according to the following criteria: (i) relevance of the indicator (ii) adequacy of the indicator in the AIDS-NGOs context (iii) ability of AIDS-NGOs to change the indicator outcome (iv) feasibility of obtaining the information needed to construct the indicator and (v) activity common to all AIDS-NGOs target group [34,35].

What new information does this new methodological phase of the study provide?

This new methodological phase, through consensus meetings between AIDS-NGO professionals and the IDIAP Jordi Gol research team, has allowed the elaboration of a Haddon matrix for each target group as well as evaluation indicators for each activity carried out by the AIDS-NGOs with regard to the objective of this activity.

### Ethical aspects

The study has been conducted according to Guidelines of the Helsinki Declaration of Good Clinical Research Practice. The project has been approved by the Ethical and Clinical Research Committee of the Institute of Research in Primary Health Care (IDIAP) Jordi Gol.

Informed Consent: The information for the study has been provided orally as well as in writing. Study subjects had sufficient opportunity to ask questions regarding details of the study. Written informed consent following the guidelines of the Helsinki Declaration was obtained.

Data confidentiality: Confidentiality and anonymity of the data have been ensured according to the Spanish law 15/1999 of data confidentiality, both in the implementation phase of the project and in presentations or publications resulting there from. Individual data was encoded to ensure anonymity. Only researchers and monitors have access to the data.

## Results

### Phase 1-Systematic and exhaustive review of the annual AIDS-NGOs reports

The objectives and activities identified using the review of the reports were aimed at 5 target populations established by the AIDS Program of Catalonia (commercial sex workers, injecting drug users, men who have sex with men, youth in high risk situations, persons living with HIV/AIDS), and 2 more identified target population established for this study (general population and women). Consequently, objectives and activities were collected into seven Haddon matrices.

**Figure 2:**
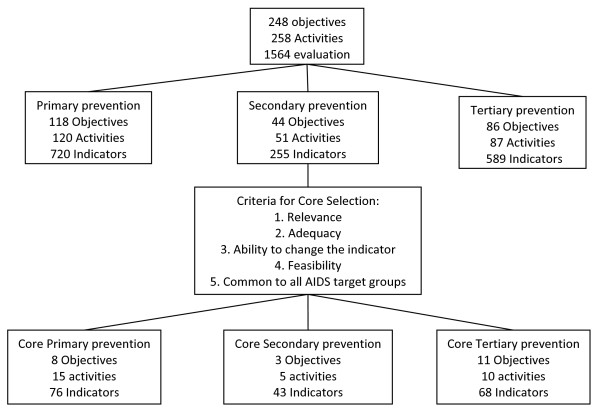
**Flow chart from the total objectives and evaluation indicators to core objectives and evaluation indicators**.

248 objectives were detected, 118 for Primary Prevention, 44 for Secondary Prevention and 86 for Tertiary Prevention (Figure 2). A total of 242 activities were identified in relation to the objectives in this phase. Some activities were similar or repeated for different objectives and different levels of prevention (Figure 2).

#### What new results were obtained?

In this phase we know the number of objectives and activities carried out by the AIDS-NGOs and the relationship between them. We also observe that many activities are common to different target groups, that different activities share the same objective, and vice versa.

### Phase 2-Transversal study

The total response to the questionnaires was 72% (26/36). Women accounted for 57.7% of the respondents.

The AIDS-NGO carried out activities for different target population. It was seen that 2/26 of the AIDS-NGOs target their activities to 7 different target groups, 12/26 to 4, 5 or 6 and 11/26 to 2 or 3 target groups.

Regarding the position of the person answering the questionnaire, "coordinator of HIV prevention activities" was the most frequent answer (15/26), followed by "person with most administrative knowledge of the AIDS-NGOs" (11/26). The category "others" was also frequently chosen (9/26). This last category included administrative personnel, managers and individuals responsible for specific programs within the AIDS-NGOs.

Regarding the question about what professionals work in AIDS-NGOs, the answer was that they have interdisciplinary teams, psychologists (64%), social workers (31%), doctors (27%), educators (23%), nurses (17%), and affected volunteers (20%) being the most frequent professional profiles.

#### What new results were obtained?

The number of identified activities increased to 258 in total, while seventeen activities (6.6%) that were not covered in the annual reports were detected. Three were especially addressed at the primary prevention level (activities that encourage closer ties with NGO services, activities that promote development leisure and interaction activities, legal advice), 3 at secondary prevention (viral load tests, risk pregnancy tests and subsequent referral, detection of tuberculosis and clinical follow-up), 5 for tertiary prevention (individualized psychological care to prison inmates, delivery of diapers to HIV+ families, websites and chats for seropositive individuals, forums and e-mail consultations and at home health care) and 6 activities present at the different levels of prevention (coordination with Spanish, European and African entities that work with HIV+ infants, adolescents and youth, guidelines to improve the doctor-patient relationship, participation in research projects, training courses and continuous education).

### Phase 3. A qualitative study with a phenomenological perspective

The main results achieved through the qualitative study were: AIDS-NGOs offered different activities adapted to the needs of the users (people who use AIDS-NGOs' services); The NGO professionals perceived that users were satisfied. The users were satisfied and felt comfortable with the education and health promotion model; the preventive activities of the AIDS-NGOs were based on a participatory health education model adjusted to the people's needs, based on empowerment and focusing on the ideas of knowledge and skills; AIDS-NGOs professionals worked in interdisciplinary groups using a holistic approach with cultural and professional competence; they were flexible, innovative and motivated; and they traveled to where the users are.

Regarding the evaluation of the AIDS-NGOs objectives and activities, the main conclusions were: evaluating the work of AIDS-NGOs is difficult (a concern voiced in most group meetings); some activities require great effort and dedication and quantitative indicators such as the number of cases are not enough to evaluate them; to improve the assessment of the activities and objectives of the AIDS-NGOs, the professionals suggest looking for quantitative and qualitative indicators that provide a reliable measure of the process and the results. More details about results in this phase have been published elsewhere [34].

#### What new information do the results of this study provide?

AIDS-NGO professionals carry out their activities on a holistic basis, maintaining confidentiality, with professional competence and cultural awareness and respecting the principle of equality. The relationship between AIDS-NGO professionals and users is based on attention, a holistic focus (both socially and in health terms), and user needs.

Users often apply to NGOs for help not only with their basic needs (food, shelter and hygiene) but also for legal aid and assistance with employment and health. Activities aimed at the prevention of HIV-AIDS infection are more effective if they take these basic needs into account. NGOs carry out specific activities aimed at promoting the accessibility of and user links with these associations. NGO professionals form flexible, motivated and innovative interdisciplinary teams ready to go wherever their users are. They require continued training and coordination between the different fields.

NGO professionals recognise the need for an adequate system for the collection of information in order to facilitate information management. They also emphasise the need to support and improve the planning and execution of the current process of the evaluation of objectives and activities. NGO professionals cite perception of a high level of satisfaction among users with regard to the attention received, as well as a lack of recognition of their activities by other professional and institutional groups.

### Phase 4. Consensus meetings between the research team and the NGO professionals

Seven Haddon matrices were developed with the results achieved in the three previous phases. In total, 1564 evaluation indicators, 720/1564 of primary prevention, 255/1564 of secondary prevention and 589/1564 of tertiary prevention were proposed (Figure 2). All the information containing the core objectives, activities and indicators are shown in detail in Additional File 1. All the indicators have been published elsewhere [36].

Finally, from all the activities and all the evaluation indicators proposed, the research team selected the core objectives and indicators based on the criteria specified above [34] as shown in table 3 and in Figure 2. Thirty core activities were undertaken: 15 for primary prevention activities (76 indicators), 5 for secondary prevention (43 indicators) and 10 for tertiary prevention (68 indicators) (Table 4).

**Table 3:** Core objectives of prevention levels by AIDS-NGO

CORE OBJECTIVES
*Primary Prevention*
Encourage the use of condoms and promotion of proper use
Increase information and prevention of HIV and other STI.
Conduct training and awareness activities for HIV/AIDS and other STI
Contribute to the improvement of the information on HIV and sexual health through various media (telephone, e-mail, internet)
Promote condom and hydro soluble lubricant use as a prevention method
Elaborate informative materials on HIV prevention
Gather written and audiovisual information on initiative studies and process experiences in HIV
Increase public awareness of the epidemic and disseminate information about AIDS
***Secondary Prevention***

Encourage the early detection of HIV/AIDS and other STI
Encourage the early detection of HIV/AIDS and other STI in the affected person's contacts
Offer medical testing services for the detection of HIV and opportunistic diseases
***Tertiary Prevention***

Ensure referral to medical services of individuals with positive diagnosis
Provide information and advice on economic, training, social and legal aid.
Provide information and advice on mental health issues
Promote human rights, equality and liberty using cultural mediation
Promote integral incorporation and autonomy in society
Develop mutual help groups of peers and encourage creation of social networks
Training of health and employment specialists
Encourage and foster support for individuals with HIV regardless of their gender
Promote and foster reintegration for seropositive individuals without economic resources
Organize events with broad social impact and including the media (combat stigmatization and achieve normalization)
Contribute to the change of attitudes on treatment adherence and safe sex

**Table 4:** Haddon matrix of core prevention-control based AIDS-NGOs activities

Prevention Level	Activities intervening on individual factors: Promoting healthy behaviors and attitudes	Intervening on factors related to the immediate	Activities aimed at intervening on factors related to the external environment: infrastructure and population	Activities aimed at intervening on factors related to the social system
**Primary Prevention**	Activities that encourage closer ties with AIDS-NGO services		Activities that encourage closer ties with NGO services	Participation in commemorative acts.
	
Health promotion and primary prevention (preventing HIV infection)	Promote and development leisure and interaction activities		Promote and development leisure and interaction activities	Participation in networking platforms and work groups related to HIV/AIDS
	
	Health education and safe sex activities: HIV information and distribution of informative materials		Health education and safe sex activities: HIV information and distribution of informative materials	Diffusion and training
	
	Educational activities to increase awareness of the male condom, the female condom, lubricants and their correct use		Educational activities to increase awareness of the male condom, the female condom, lubricants and their correct use	Training of trainers
	
	Distribution of prophylactic material: condoms and lubricants in NGO offices, saunas, etc.		Distribution of prophylactic material: condoms and lubricants in NGO offices, saunas, etc.	
	
	Distribution of informative materials on the street, in saunas, pubs, flats, clubs, schools, high schools		Distribution of informative materials on the street, in saunas, pubs, flats, clubs, schools, high schools	
	
	Personalized care and information via telephone and email		Participation in networking platforms and work groups related to HIV/AIDS	
	
	Editing and preparation of informative material for the prevention of HIV/AIDS		Training staff in peer groups	
	
	Legal advice		Legal advice	
	
	Workshops and advice on employment		Workshops and advice on employment	

**Secondary Prevention**	Counselling and rapid testing of HIV and syphilis Testing for detection of hepatitis A/B/C	Counselling and rapid testing of HIV and syphilis for the couple		
	
Measures focusing on early HIV detection	Consultations for STI detection Referrals for consultations for STI detection			
	
	Consultations for tuberculosis detection Referrals to Chest Unit for tuberculosis detection			
	
	Information and referral for post-exposure prophylaxis			

**Tertiary Prevention**	Receive health services and referrals to other health services, when necessary			
	
Attention focused on measures: reducing the effects and promote the rehabilitation reintegration	Promote the adherence to antiretroviral treatments (HAART)			
	
	Conduct emotional support sessions and advice. Conduct individual psychological therapies			
	
	Accompany individuals to medical consultations in order to improvement visits to hospitals and primary care			
	
	Give social and legal aid		Give social and legal aid	
	
	Workshops and advice on employment			
	
	Peer help groups, support groups to address HIV		Peer help groups, support groups to address HIV	
	
	Conduct alternative medicine workshops: Reiki, Bach flowers, etc.			
	
	Provide flats for HIV+ individuals regardless of their gender		Provide flats for HIV+ individuals regardless of their gender	
	
	Telephone attention, internet consultations, chats for seropositive individuals and for serodiscordant couples, forums and consultations via email			

An example of the core preventive activities and proposed evaluation indicators are described in tables 5, 6 and 7 for each level of prevention. Primary prevention indicators are mainly related to the use of barrier methods for preventing HIV infection and to the increased knowledge of HIV and Sexually Transmitted Infections (STI) (Table 5). Secondary prevention indicators are related to early detection of HIV, STI and opportunistic infections (Table 6). Tertiary prevention indicators are related with comprehensive counselling to achieve effective reintegration of individuals infected with HIV/AIDS (Table 7).

**Table 5:** Example of proposed indicators for core preventive activities at the primary prevention level

**Activities to PROMOTE THE LINK TO NGO SERVICES**. Activities to PROMOTE ACCESSIBILITY THROUGH ON-SITE CARE DURING EXTENDED HOURS			
**PROCESS MEASURE**	**Indicator description**	**Method of Data collection**	**Data Collection Frequency**

Monitoring of days public is served	Days of the year open to the public	NGO	12 months

Monitoring of schedules and days of the week that public is served	Number of hours per day and days of the week open to the public	NGO	12 months

Monitoring of hours open outside of normal schedule	Hours of the day and days of the week open outside of normal schedule	NGO	12 months

Monitoring of number of activity hours for each professional	Hours of activity for each professional (separating volunteers and part time and full time professionals)	NGO	12 months

**OUTCOME MEASURE**	**Indicator description**	**Method of Data collection**	**Data Collection Frequency**

Monitoring of the individuals attended to in one year	Number of individuals attended in one year	NGO	12 months

Monitoring of the use of the center by the same user	Number of times that the same user uses the centre per year	NGO	12 months

**Table 6:** Example of a proposed indicators for core preventive activities at the secondary prevention level

COUNSELLING AND RAPID TESTING FOR HIV AND SYPHILIS			
**PROCESS MEASURE**	**Indicator description**	**Method of Data collection**	**Data Collection Frequency**

Monitoring of various media outlets that announce the availability of the test	Number of media outlets announcing the rapid testing/Total number of media outlets used by the association	NGO	12 months

Monitoring of the number of advertisements broadcasted	Number of rapid testing advertisements per month	NGO	12 months

Monitoring number of hours of testing per week	Number of hours of testing per week/Total number of hours of activity per week	NGO	12 months

Availability of equipment and appropriate conditions to conduct rapid testing	Availability of equipment and appropriate conditions to conduct rapid testing	Direct observation	6 months

Availability of personnel trained in giving HIV/AIDS prevention advice personal	Number of professionals trained to give advice regarding rapid testing/Number of professionals that give advice	NGO	12 months

Monitoring of the number of educational materials distributed	Number of educational materials about rapid testing distributed each month	NGO	12 months

Monitoring of the number of meetings and continuing education of personnel that conduct rapid testing	Number of coordination meetings and continuing education between professionals that conduct rapid testing in order to share experiences (analysis of cases, difficulties...)	NGO	12 months

**OUTCOME MEASURE**	**Indicator description**	**Method of Data collection**	**Data Collection Frequency**

Monitoring of costs to NGOs in promoting rapid testing and the number of applications received	Total costs in the promotion of rapid testing and the number of applications received	NGO	12 months

Monitoring of individuals that solicit appointments for HIV or syphilis rapid testing	Number of individuals that solicit appointments for HIV or syphilis rapid testing, according to sex, age, marital status, and place of origin	NGO	12 months

Monitoring of the number of users that have appointments and come for rapid HIV testing	Number of users that have an appointment and finally come to get tested	NGO	12 months

Monitoring of individuals attended in pre-test, according to sex, age, marital status, sexual preference and place of origin	Number of individuals that are tested, according to sex, age, marital status and place of origin	NGO	12 months

Monitoring of tests conducted per month	Total number of rapid tests conducted per month	NGO	12 months

Monitoring of condoms distributed in CSW.	Number of condoms distributed monthly to people that receive rapid testing	NGO	12 months

Monitoring of the number of rapid testing referrals	Number of people referred to other associations for rapid testing	NGO	12 months

Monitoring of individuals that return to collect syphilis test results	Number of individuals that return to collect syphilis test results/Total number of individuals that are tested for syphilis	NGO	12 months

Monitoring of individuals that return to collect HIV test results	Number of individuals that return to collect an HIV test results/Total number of individuals that are tested for HIV	NGO	12 months

Monitoring of positive syphilis test results	Number of individuals with a positive syphilis test result/Number of individuals that return to collect syphilis test results	NGO	12 months

Monitoring of positive HIV test results	Total number of users with a positive HIV test result/Number of users that return to collect an HIV test results	NGO	12 months

Level of satisfaction with the services	Number of rapid test service users that have responded to the satisfaction questionnaire and have high, moderate or low satisfaction/Total number of rapid test service users	Users service questionnaire	12 months

**Table 7:** Example of proposed indicators for core preventive activities at the tertiary prevention level

Guarantee the follow-up and referral to medical services of individuals with a positive diagnosis			
**PROCESS MEASURE**	**Indicator description**	**Method of Data collection**	**Data Collection Frequency**

Monitoring of the number of individuals infected with HIV that have received medical care	Number of individuals infected with HIV that have received medical care/Number of individuals infected with HIV attended and that have received health care by the NGO in the evaluation year	NGO	12 months

Monitoring of the number of individuals infected with HIV that are referred to other health services	Number of individuals infected with HIV that are referred to other health services/Number of individuals infected with HIV attended and that have received health care by the NGO in the evaluation year	NGO	12 months

Monitoring of the number of individuals referred to each service	Numbers of individuals referred to each service	NGO	12 months

Monitoring of the number of people working in a service	Number of people working in a service	NGO	12 months

Monitoring of the hours dedicated to each activity	Hours dedicated to each activity	NGO	12 months

**OUTCOME MEASURE**	**Indicator description**	**Method of Data collection**	**Data Collection Frequency**

Level of satisfaction with the service	Number of individuals infected with HIV that have received care, have responded to the satisfaction questionnaire, and have a high level of satisfaction/Number of infected individuals that have received care and have responded to the questionnaire	NGO Answers to the satisfaction questionnaire	12 months

Monitoring of the questionnaire of quality of life answers of individuals attended by the NGO	Descriptive analysis and monitoring of the global score and score by dimensions of the questionnaire of quality of life answers of individuals infected with HIV and attended by the NGO	NGO Answers to the QQV	12 months

All the core evaluation indicators proposed can be seen in Additional Files 1.

Activities were carried out to achieve all of the proposed objectives by the various AIDS-NGO, although there were some objectives covered by more than one activity.

## Discussion

The present study followed a mixed method strategy that contributes to improving knowledge of activities undertaken by AIDS-NGOs and serves as a basis from which to develop a final proposal of indicators to assess these prevention-control based activities. As shown in the results, this methodological strategy implies that the different phases complement one another and progressively richer information is obtained [20,37].

The results allowed us to know all the preventive-control based activities done by AIDS-NGOs in Catalonia and how they could be assessed. The first two phases showed us a relationship between objectives and activities, and what other activities were not registered in the AIDS-NGOs annual reports. The qualitative phase showed how the preventive-control activities are done and what the main barriers and limitations to evaluate it are. Finally, the indicators consensus meetings enabled us to talk with AIDS-NGOs professionals and establish a consensus on the indicators proposed to evaluate the activities in the daily work flow.

The preventive activities of the AIDS-NGO are based on a participatory health education model adjusted to the people's needs, based on empowerment and focusing on the ideas of knowledge and skill. This education strategy follows the principles put forward by the WHO [38].

Results emphasise the importance of offering ancillary services [39] to people in need of HIV-AIDS prevention or treatment. Another study showed that the holistic person-based approach is essential to achieve a change in behaviour [40]. On the other hand, the WHO Report 2008 [38] underlines the impact of person-based care in health improvement, quality of life, user trust and treatment adherence.

In this relationship model, the professional takes into account the values and perspective of the user, and therefore incorporates them in the decision-making process [41]. The applicability of specific programmes requires a community approach to adjust them to match the needs of the target population [39,42].

The results confirm that the AIDS-NGOs perform most of their activities in relation to target groups at risk of social exclusion or because they are socially vulnerable. Moreover, it reduces social inequalities due to socioeconomic status, gender and social orientation. It also confirms the role of NGO-AIDS as a bridge acting as a "communications space" between health services and the population) and also with other services (legal, social, employment, etc.).

Evaluation is a continuous process that facilitates the identification of areas for improvement. It should also contribute to the recognition of tasks that have been satisfactorily carried out by professionals. The professionals belonging to the participating AIDS-NGOs of this study consider evaluation to be an activity which is relevant to them and share the evaluation needs. Therefore, there is concordance between the necessities detected by the HIV-AIDS program (Public Health Department) and those identified through a revision of international publications [1,43-45].

In order to evaluate the objectives and activities of AIDS-NGOs, it is essential to use good indicators bringing together characteristics such as: acceptability, feasibility, reliability, sensitivity to change, validity and meaningful and possible communicability [19,46].

The latest update of the "Compendium of Evidence-Based HIV Prevention Interventions", elaborated by the CDC, as well as the "Community guide" website, both show a broad range of evidence-based preventive activities. However, these activities are included in programs aimed at very specific groups (female condom skills training; many men, many voices; personalized cognitive risk-reduction counseling), while our study presents a broader evaluation and suggests multiple indicators to evaluate concrete activities, which can be applicable to diverse intervention programs and to different groups. Thus, this proposal of indicators can help provide new evidence about the effectiveness of specific activities according to the level of prevention and intervention [47].

Through the development of the first phase of this study, two more target groups of population were identified: inmates and immigrants. These two target populations had specific characteristics deserving of special consideration and their own preventive programs on the part of the AIDS-NGOs, and they were added as target populations for purposes of this study. Specific objectives and activities addressed to these groups were identified, and specific evaluation indicators proposed through the seven final Haddon matrices, available via the following link (http://www.gencat.cat/salut/depsalut/html/ca/dir2068/informefinal_actongsida2009.pdf) [36].

Our proposal agrees with some of the quality measures of HIV care proposed by the CDC and UNAIDS [1,45,47]. What is new and constitutes a strong point in our study is that we have added other quality indicators that measure user satisfaction (an essential aspect of the evaluation of service quality) and more psychosocial factors (legal advice) that can have an impact on the fight against inequalities and stigmatization [6,48].

Another important aspect to note is that, by using the Haddon matrix, many preventive interventions and evaluation indicators have been identified and defined on the individual, external and social levels, while less have been directed towards the individual's immediate environment. These findings are consistent with those observed by Peiró et al [12]. A possible interpretation of this phenomenon is the concern of AIDS-NGOs professionals about data confidentiality; in order to act in the immediate environment of the affected person, he or she must agree to share the problem with individuals in his or her intimate environment.

The consensus phase among AIDS-NGOs professionals and the research team to develop the indicators has meant the cooperation and participation of the professionals in this study and constitutes a key element in facilitating the acceptance and implementation of the improvements proposed in the evaluation. It is also worth mentioning that professionals belonging to the AIDS-NGOs that participated in the consensus phase make up a multidisciplinary team much like the AIDS-NGOs. This gives the proposed indicators wide variability and richness, encompassing all of the activities undertaken by the AIDS-NGOs [34,35,45].

The main limitation of this proposal of indicators is that it has not been applied. It would be important to share and disseminate it among professionals of the various programs and organizations that work with HIV in order to test its applicability, usefulness, validity and practical relevance.

A possible selection bias could also be argued, given that these indicators have been designed with the participation of professionals from AIDS-NGOs that work in Catalonia and are financed by this autonomous community's Department of Health. Nonetheless, as mentioned above, the enormous variability in the number of activities and proposed evaluation indicators minimizes this bias and raises their potential usefulness for AIDS-NGOs located in other geographical areas. Furthermore, one limitation of the study was not to have studied the views of other health providers that work with HIV-AIDS prevention or control-programmes (Primary Care, Public Health, Reproductive and Sexual Health, Faith-Based Organizations and Community Based Organizations). Although their contributions would have been of great interest, for reasons of practicality and insufficient resources this aspect could not be included.

We also want to mention that indicators for global results such as HIV mortality, rate of hospitalizations and approach to other health problems (diabetes, hypertension...) have not been proposed. However, the improvements in these epidemiological indicators can be attributed to multiple factors (improvements in security and effectiveness of drug treatments, healthcare, etc.) and not only to the AIDS-NGOs' activities. Nonetheless, studies that directly involve classic systems of epidemiological surveillance for HIV-AIDS would be of interest.

For the selection of indicators in future evaluations of preventive activities in AIDS-NGOs, the elements that make up the third dimension of the Haddon matrix should be taken into consideration: effectiveness, cost, freedom, equity, stigmatization, preferences of the affected community or individual and viability. These factors can be considered from different points of view and can have greater or lesser weight when selecting indicators, based on the general objectives of future health policies and on HIV-AIDS [12,15,16].

It is also important that the strategic plan of future HIV-AIDS policies emphasize activities that address the population that makes up the affected or at-risk person's immediate environment [12]. Although activities directed towards the social environment can have an indirect impact and their execution can be more difficult in some cases, it is necessary to emphasize the key role of the AIDS-NGOs in the development of these activities and the important social impact they can have.

## Conclusions

This study showed the importance of methodological combination for a better understanding of results and the mechanisms of changes in the evaluation of AIDS-NGO activities. Although the study had some limitations, the combination of quantitative and qualitative data gave the opportunity to construct core evaluation activities and the corresponding indicators. The results could help to homogeneously assess the preventive-control activities carried out by several AIDS-NGOs. Moreover, the indicators could help the professionals to understand and therefore to improve the evaluation of the preventive-control AIDS-NGO activities. The Haddon matrix enables us to detect the gaps in intervention levels and, as a consequence, to keep in mind strategies that could enhance AIDS programs in the future. A further important message of the research was that qualitative approaches are essential in order to identify underlying processes and to detect activity procedures in order to better define the indicators.

## Competing interests

The authors declare that they have no competing interests.

## Authors' contributions

AB, EPR, CV, AR, RM, AG and JA have been involved in writing the manuscript, and all authors critically revised and approved the final manuscript. AB, EPR, CV, AR and JA designed the methodology and contributed to the description of the background. AB, EPR, CV, AR, CA and JA have done the fieldwork and the analysis and interpretation of data. All authors have participated sufficiently in the work to take public responsibility for appropriate portions of the content.

## Pre-publication history

The pre-publication history for this paper can be accessed here:

http://www.biomedcentral.com/1472-6963/11/176/prepub

## Supplementary Material

Additional file 1 **Complete information about core objectives, activities and indicators**. NGO: Nongovernmental organizations. STI: Sexual Transmitted Diseases. QOL: Quality of life questionnaire. HAART: Highly Active Antiretroviral treatment. DO: Direct Observation. FG: Focus Groups. Click here for file
