# Supplementary material for: Core indicators evaluation of effectiveness of HIV-AIDS preventive-control programmes carried out by nongovernmental organizations. A mixed method study
Source: BMC Health Serv Res. 2011 Jul 28;11:176. doi: 10.1186/1472-6963-11-176 (PMC3155906; doi:10.1186/1472-6963-11-176)
Supplement: Additional file 1 — Complete information about core objectives, activities and indicators. NGO: Nongovernmental organizations. STI: Sexual Transmitted Diseases. QOL: Quality of life questionnaire. HAART: Highly Active Antiretroviral treatment. DO: Direct Observation. FG: Focus Groups. [file 1472-6963-11-176-S1.DOC]

**CORE ACTIVITIES, OBJECTIVES AND INDICATORS IN PRIMARY PREVENTION LEVEL**

| **Table 6.1** | | | |
| --- | --- | --- | --- |
| **Activities to PROMOTE THE LINK TO NGO SERVICES.**  **Activities to PROMOTE ACCESSIBILITY THROUGH ON-SITE CARE DURING EXTENDED HOURS** | | | |
| **Promote and develop leisrue and interaction activities** | | | |
| **PROCESS MEASURE** | **Indicator description** | **Method of Data collection** | **Data Collection Frequency** |
| Monitoring of days public is served | Days of the year open to the public | NGO | 12 months |
| Monitoring of schedules and days of the week that public is served | Number of hours per day and days of the week open to the public | NGO | 12 months |
| Monitoring of hours open outside of normal schedule | Hours of the day and days of the week open outside of normal schedule | NGO | 12 months |
| Monitoring of number of activity hours for each professional | Hours of activity for each professional (separating volunteers and part time and full time professionals) | NGO | 12 months |
| **OUTCOME MEASURE** | **Indicator description** | **Method of Data collection** | **Data Collection Frequency** |
| Monitoring of the individuals attended to in one year | Number of individuals attended in one year | NGO | 12 months |
| Monitoring of the use of the center by the same user | Number of times that the same user uses the center per year | NGO | 12 months |

| **Taula 6.2** | | | |
| --- | --- | --- | --- |
| **PROMOTE AND DEVELOP LEISURE AND INTERACTION ACTIVITIES** | | | |
| **Promote and develop leisrue and interaction activities**  **Includes activities such as: volunteer Tuesdays, snacks, movie sessions or English classes, among others. Condoms and lubricant are distributed during these activities.** | | | |
| **PROCESS MEASURE** | **Indicator description** | **Method of Data collection** | **Data Collection Frequency** |
| Monitoring of leisure and interaction activities conducted | Number of leisure and interaction activities conducted (talks, outings, snacks, movie forums, English classes) | NGO | 12 months |
| **OUTCOME MEASURE** | **Indicator description** | **Method of Data collection** | **Data Collection Frequency** |
| Monitoring of individuals that participate in each activity | Number of participants in each activity | NGO | 12 months |
| Monitoring of individuals that repeatedly participate in each | Number of individuals that repeatedly participate in each | NGO | 12 months |

| **Table 6.3** | | | |
| --- | --- | --- | --- |
| **EDUCATIONAL ACTIVITIES TO PROMOTE HEALTH AND SAFE SEX: INFORMATION ON HIV AND DISTRIBUTION OF INFORMATIVE MATERIALS ON HIV AND OTHER STI** | | | |
| **Increase information and prevention of HIV/AIDS. Conduct trainings and awareness activities on HIV/AIDS and other STI** | | | |
| **PROCESS MEASURE** | **Indicator description** | **Method of Data collection** | **Data Collection Frequency** |
| Monitoring of individual and group educational activities | Total number of individual and group activities | NGO | 12 months |
| Monitoring of individual educational activities | Number of educational activities directed to only one person | NGO | 12 months |
| Monitoring of activities in workshop format | Number of activities in workshop format | NGO | 12 months |
| Monitoring of activities in lecture format | Number of activities in lecture format | NGO | 12 months |
| Monitoring of places where education activities are conducted | Number of places where education activities are conducted (street, education centers, after school centers, high schools…) | NGO | 12 months |
| Monitoring of activities conducted | Number of activities conducted (in the street, education centers, after school centers, organizations, high schools) | NGO | 12 months |
| Monitoring of professionals that participate in each type of activity | Number of professionals that participate in each type of activity | NGO | 12 months |
| Monitoring of the duration of each activity | Average duration time of each activity | NGO | 12 months |
| Monitoring of travel time to conduct the activity | Average travel time to conduct the activity | NGO | 12 months |
| Monitoring of material distributed in each activity | Number of material distributed in each activity, differentiating between specific material and outreach material | NGO | 12 months |
| **OUTCOME MEASURE** | **Indicator description** | **Method of Data collection** | **Data Collection Frequency** |
| Monitoring of individuals participating in street activities | Number of individuals participating in street activities | NGO | 12 months |
| Monitoring of individuals participating in workshops and lectures | Number of individuals participating in workshops and lectures | NGO | 12 months |
| Monitoring of individuals participating in workshops and lectures that know: forms of transmission, prevention of HIV/AIDS | Number of individuals participating in workshops and lectures that know: forms of transmission, prevention of HIV/AIDS / Total number total of individuals participating in workshops and lectures on health education | Knowledge questionnaire | At the end of the workshop |
| Level of satisfaction of workshop participants | Number of individuals participating in educational activities and that have answered the satisfaction questionnaire and level of satisfaction/Number of participants in the workshops | Satisfaction questionnaire | At the end of the education activity |
| Direct observation of the educational activities | Direct observation (DO) of the health education safe sex activities directed to people | DO of educational activities | A few per educational activity |
| Discussion group with individuals participating in educational activities | Discussion group (DG) with individuals participating in educational activities | DG participants educational activities | A few per educational activity |

| **Table 6.4** | | | |
| --- | --- | --- | --- |
| **EDUCATIONAL ACTIVITIES TO INCREASE AWARENESS OF THE MALE CONDOM, FEMALE CONDOM, LUBRICANTES AND THEIR CORRECT USE** | | | |
| **Encourage the use of the male condom, the female condom, lubricants and the promotion of their correct use.** | | | |
| **PROCESS MEASURE** | **Indicator description** | **Method of Data collection** | **Data Collection Frequency** |
| Monitoring of the preparation time for educational activities and the time for coordination meetings between professionals | Average time of preparation and coordination for each educational activity | NGO | 12 months |
| Monitoring of individual and group educational activities | Total number of individual and group educational activities | NGO | 12 months |
| Monitoring of individual educational activities | Number of individual educational activities directed to one individual | NGO | 12 months |
| Monitoring of activities in workshop format | Number of activities in workshop format | NGO | 12 months |
| Monitoring of activities in lecture format | Number of activities in lecture format | NGO | 12 months |
| Monitoring of places where education activities are conducted | Number of places where education activities are conducted (street, education centers, after school centers, high schools…) | NGO | 12 months |
| Monitoring of activities conducted | Number of activities conducted (in the street, education centers, after school centers, organizations, high schools) | NGO | 12 months |
| Monitoring of professionals that participate in each type of activity | Number of professionals that participate in each type of activity | NGO | 12 months |
| Monitoring of the duration of each activity | Average duration time of each activity | NGO | 12 months |
| Monitoring of travel time to conduct the activity | Average travel time to conduct the activity | NGO | 12 months |
| **OUTCOME MEASURE** | **Indicator description** | **Method of Data collection** | **Data Collection Frequency** |
| Monitoring of participants in workshops | Number of participants in workshops | NGO | 12 months |
| Monitoring of participants in lectures | Number of participants in lectures | NGO | 12 months |
| Monitoring of participants in street activities | Number of participants in street activities | NGO | 12 months |
| Monitoring of participants that know about correct use of preventive materials | Number of participants that know about correct use of preventive materials male condoms, female condoms, lubricants/ Total number of participants that have participated in workshops and lectures | NGO  Knowledge questionnaire | At the end of the workshop |
| Level of satisfaction with workshops | Number of participants in educational activities and that have answered the satisfaction questionnaire and level of satisfaction/Number of participants in the workshops | NGO  Satisfaction questionnaire | At the end of the education activity |
| Direct observation (DO) of the educational activities | DO of the educational activities | OD de les AE | A few per educational activity |
| Discussion group (DG) with participants of educational activities | DG participants of educational activities | GD amb participants AE | A few per educational activity |

| **Table 6.5** | | | |
| --- | --- | --- | --- |
| **DISTRIBUTION OF PROPHYLACTIC MATERIAL: CONDOMS, LUBRICANTS, MATERIALS FOR NGOS, SAUNAS…** | | | |
| **Encourage the use of the male condom, the female condom, lubricants and the promotion of their correct use.** | | | |
| **PROCESS MEASURE** | **Indicator description** | **Method of Data collection** | **Data Collection Frequency** |
| Description of distribution channels | Description of distribution channels | NGO | 12 months |
| Monitoring of streets, saunas, pubs, clubs where prophylactic material is distributed | Number of streets, saunas, pubs, clubs where male condoms, female condoms and lubricants are distributed | NGO | 12 months |
| Monitoring of NGO staff that participate actively in the activities | Number of NGO staff that participate actively in each activity | NGO | 12 months |
| Monitoring of the number of times that the activities are repeated | Number of times that an activity is repeated in each location | NGO | 12 months |
| Monitoring of the number days/times per week that activities are repeated | Number of days/times per week that activities are held | NGO | 12 months |
| **OUTCOME MEASURE** | **Indicator description** | **Method of Data collection** | **Data Collection Frequency** |
| Monitoring of male condoms distributed in each place | Number of male condoms distributed in the street, saunas, pubs, flats, clubs... | NGO | 12 months |
| Monitoring of female condoms distributed in each place | Number of female condoms distributed in the street, saunas, pubs, flats, clubs... | NGO | 12 months |
| Monitoring of lubricant distributed in each place | Number of lubricant distributed in the street, saunas, pubs, flats, clubs...… | NGO | 12 months |

| **Table 6.6** | | | |
| --- | --- | --- | --- |
| **DISTRIBUTION OF INFORMATIVE MATERIALS TO THE OWNERS OF BARS, PUBS, FLATS, CLUBS** | | | |
| **Increase information and prevention of HIV/AIDS and other STI** | | | |
| **PROCESS MEASURE** | **Indicator description** | **Method of Data collection** | **Data Collection Frequency** |
|  | Description of:   - Type of informative material disbributed - Addressed populations - Content - Distriubtion channels - Origin (developed by NGO or others) | NGO | 12 months |
| Monitoring of streets, saunas, pubs, clubs where prophylactic material is distributed | Number of streets, saunas, pubs, clubs,... where informative material is distributed | NGO | 12 months |
| Monitoring of NGO staff that participate actively in the activities | Number of NGO staff that participate actively in each activity | NGO | 12 months |
| Monitoring of the number of times that the activities are repeated | Number of times that an activity is repeated in each location | NGO | 12 months |
| Monitoring of travel time to conduct the activity | Average travel time to distribuye material | NGO | 12 months |
| **OUTCOME MEASURE** | **Indicator description** | **Method of Data collection** | **Data Collection Frequency** |
| Monitoring of informative materials distributed in each place | Number of informative materials distributed in in the street, saunas, pubs, flats, clubs according to content | NGO | 12 months |

| **Table 6.7** | | | |
| --- | --- | --- | --- |
| **EDITING AND PREPARATION OF INFORMATIVE MATERIAL FOR HIV AND STI PREVENTION. CATALOGING OF WRITTEN, AUDIOVISUAL AND COMPUTERIZED INFORMATION RELATED TO HIV AND STI IN ORDER TO HAVE AN ARCHIVE OF DOCUMENTS WITH EASY ACCESS AND UPDATING** | | | |
| **Contribute to improving information on AIDS: treatments, disesases, secondary effects, safe sex** | | | |
| **PROCESS MEASURE** | **Indicator description** | **Method of Data collection** | **Data Collection Frequency** |
| Monitoring of materials prepared for diffusion to NGOs | Number of materials prepared for diffusion to NGOs | NGO | 12 months |
| Monitoring Monitoring of materials on HIV and STI prevention and information prepared for diffusion | Number of materials on HIV and STI prevention and information prepared for diffusion | NGO | 12 months |
| Monitoring of preparation process of all informative material | Describe the preparation process of informative material:   - Target population, - Participation of the target population - collaborative prepation (Generalitat, NGO and users...) - pilot test to evaluate: comprehension, practical usefulness, form (colors, images…), clarity of content/messages | Specific study |  |
| Monitoring of informative materials or search services offered by the cataloging center | Number and types of informative materials or search services offered by the cataloging center (pamphlets, books, magazines, website information, bibliographic search services…) | NGO | 12 months |
| Monitoring of the date of the last update of material | Date of the last update of material in the archive and catalog fons | NGO | 12 months |
| **OUTCOME MEASURE** | **Indicator description** | **Method of Data collection** | **Data Collection Frequency** |
| Monitoring of the number of inquiries in the catalog center by NGOs | Number of inquiries in the catalog center by NGOs | NGO | 12 months |
| Monitoring of the number of entrances or connections to the catalog crenter´s website | Number of entrances or connections to the catalog crenter´s website | NGO | 12 months |
| Monitoring of the number of users that have used the catalog center´s website to obtain inofmration about HIV and STI. | Number of users that have used the catalog center´s website to obtain information about HIV and STI. Evaluation by in depth interviews or an ad-hoc questionnaire to measure the impact of the informative material   1. Do they know of the material? 2. Have they looked at it? 3. Do they have it, or have they had it, at home? 4. Opinions on the form and content? 5. Does it reach the objectives? | NGO  In depth interviews  Satisfaction questionnaire | 12 months |
| Level of user satisfaction | Number of people that have used information in the catalog center and have responded to the satisfaction questionnaire and level of satisfaction/ Number of people that have used information in the catalog center | NGO  Satisfaction questionnaire | 12 months |

| **Table 6.8** | | | |
| --- | --- | --- | --- |
| **TRAINING ACTIVITIES TO EDUCATION PEER GROUPS** | | | |
| **Enhance mutual, peer help groups and encourage the creation of social networks** | | | |
| **PROCESS MEASURE** | **Indicator description** | **Method of Data collection** | **Data Collection Frequency** |
| Monitoring of training workshops for educators of peer groups | Number of training workshops for educators of peer groups according to characteristics (sex, age, sexual orientation, origin, association) | NGO | 12 months |
| **OUTCOME MEASURE** | **Indicator description** | **Method of Data collection** | **Data Collection Frequency** |
| Monitoring of the learning process between educators of peer groups | Number of people that have received training as peer group educators and have obtained a high score in the knowledge test/ / Number of people that have received training as peer group educators | NGO  Knowledge change questionnaire | 12 months |
| Monitoring of education activities | Number of people that have received training and conduct peer group educational activities | NGO | 12 months |
| Monitoring of the level of satisfaction with trainings | Number of people that have received training as peer group educators and have responded to the satisfaction questionnaire and the level of satisfaction/ Number of people that have received education in peer groups | NGO  Satisfaction questionnaire | 12 months |

| **Table 6.9** | | | |
| --- | --- | --- | --- |
| **ACTIVITIES TO PROMOTE ACCESSIBILITY AND PERSONALIZED CARE BY 24 HOUR TELEPHONE SERVICE** | | | |
| **Contribute to the improvement of information on HIV and sexual health by telephone** | | | |
| **PROCESS MEASURE** | **Indicator description** | **Method of Data collection** | **Data Collection Frequency** |
| Monitoring of the hours of telephone service | Total number of hours dedicated to personalized service via telephone. | NGO | 12 months |
| **OUTCOME MEASURE** | **Indicator description** | **Method of Data collection** | **Data Collection Frequency** |
| Monitoring of people that have received personalized service via telephone | Number of people that have received personalized service via telephone | NGO | 12 months |
| Monitoring of the duration of telephone consultations (minutes) | Average duration of telephone consultations in minutes | NGO | 12 months |
| Level of satisfaction with personalized telephone service | Number of people or clients that have responded to the satisfaction questionnaire and level of satisfaction/ Number of people that have used personalized the telephone service | NGO  Satisfaction questionnaire | 12 months |

| **Table 6.10** | | | |
| --- | --- | --- | --- |
| **MAINTAINING CONTACT WITH EDUCATORS THROUGH E-MAIL OR INTERNET** | | | |
| **Contribute to the improvement of information on HIV and sexual health through various media (telephone, e-mail, internet)** | | | |
| **PROCESS MEASURE** | **Indicator description** | **Method of Data collection** | **Data Collection Frequency** |
| Monitoring of daily service hours via e-mail and Internet | Total number of hours dedicated to personalized service through e-mail and Internet | NGO | 12 months |
| **OUTCOME MEASURE** | **Indicator description** | **Method of Data collection** | **Data Collection Frequency** |
| Monitoring of people that have received personalized service via e-mail | Number of people that have received personalized service via e-mail | NGO | 12 months |
| Monitoring the time in minutes to respond | Average duration to respond (in minutes) | NGO | 12 months |
| Monitoring of the time between the consultation and the response | Time in days between the consultation and the response | NGO | 12 months |
| Monitoring of people that have received personalized service via Internet | Number of people that have received personalized service via Internet | NGO | 12 months |
| Monitoring of the amount of time consumed in answering consultations via e-mail o Internet | Mean answer time in minutes | NGO | 12 months |
| Level of satisfaction with personalized service via e-mail or Internet | Number of people or clients that have responded to the satisfaction questionnaire and level of satisfaction/ Number of people that have received on-site services | NGO  Satisfaction questionnaire | 12 months |

| **Table 6.11** | | | |
| --- | --- | --- | --- |
| **PARTICIPATION IN COMMEMORATIVE ACTS: AIDS MEMORIAL (MAY), TESTING DAY (OCTOBER) AND WORLD AIDS DAY** | | | |
| **Increase public awareness of the epidemic and dissemination of information about HIV/AIDS** | | | |
| **PROCESS MEASURE** | **Indicator description** | **Method of Data collection** | **Data Collection Frequency** |
| Description and monitoring of activities conducted on each commemorative day | Number of activities conducted on each commemorative day and description of the activities. | NGO | 12 months |
| Monitoring of participants in the commemorative acts | Number of attendees in each NGO activity*. | NGO | 12 months |
| Monitoring of each NGO´s media participation | Number of media participations (press, magazines, TV, radio) for each NGO | NGO | 12 months |
| Monitoring of collaborations established with other NGOs. | Number of collaborations established with other NGOs | NGO | 12 months |

**CORE ACTIVITIES, OBJECTIVES AND INDICATORS IN SECONDARY PREVENTION LEVEL**

| **Table 7.1** | | | |
| --- | --- | --- | --- |
| **CONUNSELLING AND RAPID TESTING FOR HIV AND SYPHILIS** | | | |
| **Encourage the early detection of HIV and other STI** | | | |
| **PROCESS MEASURE** | **Indicator description** | **Method of Data collection** | **Data Collection Frequency** |
| Monitoring of various media outlets that announce the availability of the test | Number of media outlets announcing the rapid testing/ Total number of media outlets used by the association | NGO | 12 months |
| Monitoring of the number of advertisements broadcasted | Number of rapid testing advertisements per month | NGO | 12 months |
| Monitoring number of hours of testing per week | Number of number of hours of testing per week / Total number of hours of activity per week | NGO | 12 months |
| Availability of equipment and appropriate conditions to conduct rapid testing | Availability of equipment and appropriate conditions to conduct rapid testing | Direct observation | 6 months |
| Availability of personnel trained in giving HIV/AIDS prevention advice personal | Number of professionals trained to give advice regarding rapid testing/ Number of professionals that give advice | NGO | 12 months |
| Monitoring of the number of educational materials distributed | Number number of educational materials about rapid testing distributed each month | NGO | 12 months |
| Monitoring of the number of meetings and continuing education of personnel that conduct rapid testing | Number of coordination meetings and continuing education between professionals that conduct rapid in order to share experiences (analysis of cases, difficulties…) | NGO | 12 months |

| **COUNSELLING AND RAPID TESTING FOR HIV AND SYPHILIS** | | | |
| --- | --- | --- | --- |
| **Encourage the early detection of HIV and other STI.** | | | |
| **OUTCOME MEASURE** | **Indicator description** | **Method of Data collection** | **Data Collection Frequency** |
| Monitoring of costs to NGOs in promoting the rapid test and the number of applications received | Total costs in the promotion of the raid test and the number of applications received | NGO | 12 months |
| Monitoring of individuals that solicit appointments for HIV or syphilis rapid testing | Number of individuals that solicit appointments for HIV or syphilis rapid testing, according to sex, age, marital status, and place of origin | NGO | 12 months |
| Monitoring of the number of users that have an appointments and come for rapid HIV testing | Numberof users that have an appointment and finally come to get tested | NGO | 12 months |
| Monitoring of individuals attended in pre-test, according to sex age, marital status, sexual preference and place of origin | Number of individuals that are tested, according to sex, age, marital status and place of origin | NGO | 12 months |
| Monitoring of tests conducted per month | Total number of rapid tests conducted per month | NGO | 12 months |
| Monitoring of condoms distributed in CSW. | Number of condoms distributed monthly to people that receive rapid testing | NGO | 12 months |
| Monitoring of the number of rapid testing referrals | Number of people referred to other associations for rapid testing | NGO | 12 months |
| Monitoring of individuals that return to collect syphilis test results | Number of individuals that return to collect syphilis test results /  Total number of indivdiuals that are tested for syphilis | NGO | 12 months |
| Monitoring of individuals that return to collect HIV test results | Number of individuals that return to collect an HIV test results /  Total number of indivdiuals that are tested for HIV | NGO | 12 months |
| Monitoring of positive syphilis test results | Number of individuals with a positive syphilis test result/  Number of individuals that return to collect syphilis test results | NGO | 12 months |
| Monitoring of positive HIV test results | Total number of users with a positive HIV test result/ Number of users that return to collect an HIV test results | NGO | 12 months |
| Level of satisfaction with the services | Number of rapid test service users that have responded to the satisfaction questionnaire and have high, moderate or low satisfaction/ Total number of rapid test service users | Users service questionnaire | 12 months |

| **Table 7.2** | | | |
| --- | --- | --- | --- |
| **COUNSELLING AND CONDUCTING HEPATITIS A/B/C* DETECTION TEST** | | | |
| **Encourage the early detection of HIV and other STI.** | | | |
| **OUTCOME MEASURE** | **Indicator description** | **Method of Data collection** | **Data Collection Frequency** |
| Monitoring of individuals that are tested for hepatitis A/B/C | Number of individuals that have had the hepatitis A/B/C detection test according to age and place of origin per month | NGO | 12 months |
| Monitoring of condoms distributed monthly to people that receive the hepatitis detection test | Number of condoms distributed monthly to people that are tested for hepatitis A/B/C | NGO | 12 months |
| Monitoring of individuals that receive advice prior to hepatitis testing | Number of individuals that receive advice prior to hepatitis testing / Total number of Number total individuals that receive the hepatitis A/B/C detection test | NGO | 12 months |
| Monitoring of individuals that return to collect hepatitis test results | Numbe of individuals that return to collect hepatitis test results / Total number of indivdiuals that are tested for hepatitis A/B/C | NGO | 12 months |
| Monitoring of positive hepatitis test results | Total number of positive hepatitis test results / Total number of individuals that are tested for hepatitis A/B/C | NGO | 12 months |
| Monitoring of individuals with negative hepatitis test results that receive the first vaccine dosis | Number of individauls with negative hepatitis test results that receive the first vaccine dosis / Total number of negative hepatitis test results | NGO | 12 months |
| Monitoring of individuals that receive the first dosis of the hepatitis vaccine that follow- up with the subsequent dosis | of individuals that receive the first dosis of the hepatitis vaccine that follow- up with the subsequent dosis (2^nd^ and 3^rd^ dosis) | NGO | 12 months |
| Monitoring of individuals with analytic follow-up after hepatitis vaccination | Number of individuals with analytic follow-up after hepatitis vaccination | NGO | 12 months |
| Level of satisfaction with the service | Number of individuals that have responded to the satisfaction questionnaire and level of satisfaction/Number of individuals attended to by the NGO that has received advice and/or have been tested | NGO  Satisfaction questionnaire | 12 months |
| Monitoring of time dedicated to the activity and to its prepration | Hours dedicated to the activity and to its prepration | NGO | 12 months |

| **Table 7.3** | | | |
| --- | --- | --- | --- |
| **REFERRALS FOR THE HEPATITIS A/B/C DETECTION TEST** | | | |
| **Encourage the early detection of HIV and other STI.** | | | |
| **PROCESS MEASURE** | **Indicator description** | **Method of Data collection** | **Data Collection Frequency** |
| Monitoring of advice on hepatitis A/B/C diagnosis | Number of advice sessions given on hepatitis A/B/C | NGO | 12 months |
| Monitoring of referrals for hepatitis A/B/C diagnosis | Number of referrals for hepatitis A/B/C diagnosis | NGO | 12 months |
| **OUTCOME MEASURE** | **Indicator description** | **Method of Data collection** | **Data Collection Frequency** |
| Monitoring of individuals that have had the hepatitis A/B/C diagnosis test | Number of individuals referred that have had the hepatitis A/B/C diagnosis test | NGO | 12 months |

| **Table 7.4** | | | |
| --- | --- | --- | --- |
| **CONSULTATIONS FOR STI DETECTION** | | | |
| **Encourage the early detection of HIV and other STI.** | | | |
| **OUTCOME MEASURE** | **Indicator description** | **Method of Data collection** | **Data Collection Frequency** |
| Monitoring of individuals that have had consultations for STI detection | Number of individuals that have had consultations for STI detection according to age and place of origin, per month | NGO | 12 months |
| Monitoring of condoms distributed to individuals that receive STI advice | Number of condoms distributed to individuals that receive STI advice per month | NGO | 12 months |
| Monitoring of individuals that receive STI detection tests | Total number of individuals that are tested for STIs | NGO | 12 months |
| Monitoring of users that return to collect STI detection test results | Number of users that return to collect STI detection test results /Tota numberl of individuals that receive STI detection tests | NGO | 12 months |
| Monitoring of users that take their partner to get tested for STIs | Total number of users that take their partner to get tested for STIs / Total number of individuals that are tested | NGO | 12 months |
| Level of satisfaction with services | Number of individuals that have responded to the satisfaction questionnaire and level of satisfaction/Number of individuals attended to by the NGO that have received advice and/or have been tested for STIs | NGO  Satisfaction questionnaire | 12 months |

| **Table 7.5** | | | |
| --- | --- | --- | --- |
| **PARTICIPATION IN TESTING DAY (OCTOBER)** | | | |
| **Increase public awareness of the epidemic and dissemination of information about HIV/AIDS** | | | |
| **PROCESS MEASURE** | **Indicator description** | **Method of Data collection** | **Data Collection Frequency** |
| Description and monitoring of activities conducted on testing day | Number monitoring of activities conducted on testing day and their description | NGO | 12 months |
| Monitoring of the participants in testing day commemorative acts | Number of attendees at NGO testing day activities | NGO | 12 months |
| Monitoring of each NGO´s media participation | Number of media participations (press, magazines, TV, radio) for each NGO on testing day | NGO | 12 months |
| Monitoring of collaborations established with other NGOs | Number of collaborations established with other NGOs | NGO | 12 months |

**CORE ACTIVITIES, OBJECTIVES AND INDICATORS IN TERTIARY PREVENTION LEVEL**

| **Table 8.1** | | | |
| --- | --- | --- | --- |
| **FOLLOW-UP AND REFERRAL TO MEDICAL SERVICES OF INDIVIDUALS WITH A POSITIVE DIAGNOSIS** | | | |
| **Guarantee the follow-up and referral to medical services of individuals with a positive diagnosis** | | | |
| **PROCESS MEASURE** | **Indicator description** | **Method of Data collection** | **Data Collection Frequency** |
| Monitoring of the number of individuals infected with HIV that have received medical care | Number of individuals infected with HIV that have received medical care / Number of individuals infected with HIV attended and that have received health care by the NGO in the In the evaluation year | NGO | 12 months |
| Monitoring of the number of individuals infected with HIV that are referred to other health services | Number of individuals infected with HIV that are referred to other health services / Number of individuals infected with HIV attended and that have received health care by the NGO in the In the evaluation year | NGO | 12 months |
| Monitoring of the number of individuals referred to each service | Numbers of individuals referred to each service | NGO | 12 months |
| Monitoring of the number of people working in a service | Number of people working in a service | NGO | 12 months |
| Monitoring of the hours dedicated to each activity | Hours dedicated to each activity | NGO | 12 months |
| **OUTCOME MEASURE** | **Indicator description** | **Method of Data collection** | **Data Collection Frequency** |
| Level of satisfaction with the service | Number of individuals infected with HIV that have received care, have responded to the satisfaction questionnaire, and have a high level of satisfaction/Number of infected individuals that have received care and have responded to the questionnaire | NGO  Answers to the satisfaction questionnaire | 12 months |
| Monitoring of the QOL answers of individuals attended by the NGO | Descriptive analysis and monitoring of the global score and score by dimensions of the QOL answers of individuals infected with HIV and attended by the NGO | NGO  Answers to the QOL | 12 months |

| **Table 8.2** | | | |
| --- | --- | --- | --- |
| **PROMOTE ADHERENCE TO TREATMENTS AND ANTIRETROVIRALS (HAART)** | | | |
| **Guarantee and improve adherence to antiretroviral treatment** | | | |
| **PROCESS MEASURE** | **Indicator description** | **Method of Data collection** | **Data Collection Frequency** |
| Monitoring of the number of individuals infected with HIV that receive HAART | Number of individuals infected with HIV that receive HAART /Number of individuals with advanced HIV infection | NGO | 12 months |
| Monitoring of the number of individuals that are in HAART | Numbers of individuals that are in HAART | NGO | 12 months |
| Monitoring of the number of individuals infected with HIV that receive HAART and that continue treatment | Number of individuals infected with HIV that receive HAART and that continue treatment at 6, 12 and 24 months | NGO | 12 months |
| Monitoring of the number of individuals that work with the activity | Individuals that work with the activity | NGO | 12 months |
| Monitoring of the number of hours dedicated to the activity | Number of hours dedicated to the activity | NGO | 12 months |
| **OUTCOME MEASURE** | **Indicator description** | **Method of Data collection** | **Data Collection Frequency** |
| Monitoring of the QOL scores of people attended by the NGO | Descriptive analysis and monitoring of the global score and score by dimensions of the QOL answers of individuals infected with HIV and attended by the NGO | NGO  Answers to QOL | 12 months |

| **Table 8.3** | | | |
| --- | --- | --- | --- |
| **CONDUCTING EMOTIONAL SUPPORT SESSIONS AND GIVING ADVICE**  **CONDUCTING INDIVIDUAL PSYCHOLOGICAL THERAPY** | | | |
| **Contribute to improving quality of life of affected individuals**  **Provide information and advice on mental health issues** | | | |
| **PROCESS MEASURE** | **Indicator description** | **Method of Data collection** | **Data Collection Frequency** |
| Monitoring of the number of individuals infected with HIV that have received emotional support and advice | Number of individuals infected with HIV that have received emotional support and advice/ Number of individuals infected with HIV attended by the NGO in the evaluation year | NGO | 12 months |
| Monitoring of the number of individuals infected with HIV that have received individual psychological therapy | Number of individuals infected with HIV that have received individual psychological therapy / Number of individuals infected with HIV attended by the NGO in the evaluation year | NGO | 12 months |
| Monitoring of the number of individuals that work with the activity | Individuals that work with the activity | NGO | 12 months |
| Monitoring of the number of hours dedicated to the activity | Number of hours dedicated to the activity | NGO | 12 months |
| Monitoring of the number of hours and number of professionals | Number of hours and number of professionals | NGO | 12 months |
| **OUTCOME MEASURE** | **Indicator description** | **Method of Data collection** | **Data Collection Frequency** |
| Level of satisfaction with the activity | Number of individuals infected with HIV that have received emotional support and advice, have responded to the satisfaction questionnaire and have a high level of satisfaction / Number of individuals infected with HIV that have received emotional support and advice and have responded to the satisfaction questionnaire | NGO  Answers to the satisfaction questionnaire | 12 months |
| Monitoring of the QOL scores of people attended by the NGO | Descriptive analysis and monitoring of the global score and score by dimensions of the QOL answers of individuals infected with HIV and attended by the NGO | NGO  Answers to the QOL | 12 months |

| **Table 8.4** | | | |
| --- | --- | --- | --- |
| **ACCOMPANYING INDIVIDUALS TO MEDICAL VISITS IN ORDER TO STREAMLINE VISITS TO THE HOSPITAL, PC** | | | |
| **Offer accompanying and human support to individuals infected with HIV and to their family members during the entire process of the disease .** | | | |
| **PROCESS MEASURE** | **Indicator description** | **Method of Data collection** | **Data Collection Frequency** |
| Monitoring of the number of individuals infected with HIV that have been accompanied to medical visits, home care or various tasks | Number of individuals infected with HIV that have been accompanied to medical visits, home care or various tasks / Number of individuals infected with HIV attended by the NGO in the evaluation year | NGO | 12 months |
| Monitoring of the number of hours dedicated to accompanying to medical visits, to home care or other tasks | of hours dedicated to accompanying to medical visits, to home care or other tasks  / Number of hours dedicated to individuals with HIV attended by the NGO in the evaluation year | NGO | 12 months |
| Monitoring of the number of individuals that work with the activity | Individuals that work with the activity | NGO | 12 months |
| Monitoring of the number of hours dedicated to the activity | Number of hours dedicated to the activity | NGO | 12 months |
| **OUTCOME MEASURE** | **Indicator description** | **Method of Data collection** | **Data Collection Frequency** |
| Level of satisfaction with the activity | Number of individuals infected with HIV that have been accompanied to medical visits, home care or various tasks, have responded to the satisfaction questionnaire and have a high level of satisfaction / Number of individuals infected with HIV that have that have been accompanied to medical visits, home care or various tasks and have responded to the satisfaction questionnaire | NGO  Answers to the satisfaction questionnaire | 12 months |
| Monitoring of the QOL scores of people attended by the NGO | Descriptive analysis and monitoring of the global score and score by dimensions of the QOL answers of individuals infected with HIV and attended by the NGO | NGO  Answers to the QOL | 12 months |

| **Table 8.5** | | | |
| --- | --- | --- | --- |
| **SOCIAL AND LEGAL SUPPORT** | | | |
| **Provide information and advice on economic, training and legal support for seropositive individuals** | | | |
| **PROCESS MEASURE** | **Indicator description** | **Method of Data collection** | **Data Collection Frequency** |
| Monitoring of the number of individuals infected with HIV that have received social support | Monitoring of the number of individuals infected with HIV that have received social support / Number of individuals infected with HIV attended by the NGO in the evaluation year | NGO | 12 months |
| Monitoring of the number of individuals infected with HIV that have received legal support | Number of individuals infected with HIV that have received legal support / Number of individuals infected with HIV attended by the NGO in the evaluation year | NGO | 12 months |
| **OUTCOME MEASURE** | **Indicator description** | **Method of Data collection** | **Data Collection Frequency** |
| Level of satisfaction with the activity | Number of individuals infected with HIV that have received support, have responded to the satisfaction questionnaire and have a high level of satisfaction / Number of individuals infected with HIV that have received support and have responded to the satisfaction questionnaire | NGO  Answers to the satisfaction questionnaire | 12 months |
| Descriptive analysis and monitoring of QOL answers of individuals attended by the NGO | Descriptive analysis and monitoring of the global score and score by dimensions of the QOL answers of individuals infected with HIV and attended by the NGO | NGO  Answers to the quality of life questionnaire | 12 months |

| **Table 8.6** | | | |
| --- | --- | --- | --- |
| **MUTUAL HELP GROUPS, SUPPORT GROUPS TO ADDRESS HIV (GAM)** | | | |
| **Contribute to mutual support of users and mutual solidarity.Promote peer groups .** | | | |
| **PROCESS MEASURE** | **Indicator description** | **Method of Data collection** | **Data Collection Frequency** |
| Monitoring of the number of individuals that have participated in mutual help support groups to address HIV | Number of individuals that have participated in mutual help support groups to address HIV | NGO | 12 months |
| Monitoring of the number of mutual help groups conducted in one year | Number of mutual help groups conducted in one year | NGO | 12 months |
| Monitoring of the number of meetings for each group | Number of meetings for each group | NGO | 12 months |
| Monitoring of the number of people that work with the activity | People that work with the activity | NGO | 12 months |
| Monitoring of the number of total hours dedicated to the activity | Number of total hours dedicated to the activity | NGO | 12 months |
| **OUTCOME MEASURE** | **Indicator description** | **Method of Data collection** | **Data Collection Frequency** |
| Number of individuals that have used the mutual hlp groups, have responded to the satisfaction questionnaire and have a high level of satisfaction / Number of individuals that have used the mutual hlp groups and have responded to the satisfaction questionnaire | Number of individuals that have used the mutual hlp groups, have responded to the satisfaction questionnaire and have a high level of satisfaction / Number of individuals that have used the mutual hlp groups and have responded to the satisfaction questionnaire | NGO  Anonymous user service questionnaire | 12 months |
| Descriptive analysis and monitoring of QOL answers of individuals infected with HIV attended by the NGO | Descriptive analysis and monitoring of the global score and score by dimensions of the QOL answers of individuals infected with HIV and attended by the NGO | NGO  Answers to the quality of life questionnaire | 12 months |

| **Table 8.7** | | | |
| --- | --- | --- | --- |
| **WORKSHOPS AND ADVICE ON EMPLOYMENT** | | | |
| **Promote integral incorporation and autonomy in society.**  **Promote and foster reinsertion for sero positive individuals without economic resources** | | | |
| **PROCESS MEASURE** | **Indicator description** | **Method of Data collection** | **Data Collection Frequency** |
| Monitoring the number of pre-employment training workshops conducted and cultural mediation | Number of pre-employment training workshops conducted and cultural mediation | NGO | 12 months |
| Monitoring of the number of advice sessions to facilitate tools and resources for finding work per person with a work permit | Number of advice sessions to facilitate tools and resources available to help find work | NGO | 12 months |
| Monitoring of the number of advice sessions for people without work permits in order to follow-up their employment itinerary and to advise how they can obtain a work permit | Number of advice sessions for people without work permits in order to follow-up their employment itinerary and to advise how they can obtain a work permit | NGO | 12 months |
| Monitoring of the number of referrals to centers and resources based on user demands | Number of referrals to centers and resources based on user demands: adult training schools, general training, etc. | NGO | 12 months |
| Monitoring of the number of people that work with the activity | People that work with the activity | NGO | 12 months |
| Monitoring of the number of hours dedicated to the activity | Number of hours dedicated to the activity | NGO | 12 months |
| **OUTCOME MEASURE** | **Indicator description** | **Method of Data collection** | **Data Collection Frequency** |
| Level of satisfaction with the activity | Number of individuals that have used employment advice services and workshops, have responded to the satisfaction questionnaire and have a high level of satisfaction / Number of individuals that have used employment advice services and workshops and have responded to the satisfaction questionnaire | NGO  Anonymous user service questionnaire | 12 months |
| Descriptive analysis and monitoring of QOL answers of individuals attended by the NGO | Descriptive analysis and monitoring of the global score and score by dimensions of the QOL answers of individuals infected with HIV and attended by the NGO | NGO  Answers to the quality of life questionnaire | 12 months |

| **Table 8.8** | | | |
| --- | --- | --- | --- |
| **COMPLEMENTARY MEDICINE WORKSHOPS: REIKI, BACH FLOWERS, ETC.** | | | |
| **Promote complentary medicine sessions in order to improve physical and psychological well being of HIV+ individuals** | | | |
| **PROCESS MEASURE** | **Indicator description** | **Method of Data collection** | **Data Collection Frequency** |
| Monitoring the number of alternative medicine sessions conducted | Number of alternative medicine sessions conducted per day   1. reiki 2. Bach flowers 3. massage 4. foot reflexology workshops 5. Shiatsu 6. Yoga nd TaiChi | NGO | 12 months |
| Monitoring of participants in each alternative medicine session | Number of participants in each alternative medicine session. | NGO | 12 months |
| Monitoring of participants in that repeat alternative medicine sessions | Number of participants in that repeat alternative medicine sessions | NGO | 12 months |
| Monitoring of the number of hours of each session | Number of hours of each session | NGO | 12 months |
| Monitoring of the total number of hours for each workshop | Total number of hours for each workshop (preparation, workshops and coordination) | NGO | 12 months |
| Monitoring of the number of people at the NGO that work with the activity | People at the NGO that work with the activity | NGO | 12 months |
| **OUTCOME MEASURE** | **Indicator description** | **Method of Data collection** | **Data Collection Frequency** |
| Level of satisfaction with the activity | Number of individuals that have used alternative medicine workshops, have responded to the satisfaction questionnaire and have a high level of satisfaction / Number of individuals that have used alternative medicine workshops and have responded to the satisfaction questionnaire | NGO  Anonymous user service questionnaire | 12 months |
| Descriptive analysis and monitoring of QOL answers of individuals attended by the NGO | Descriptive analysis and monitoring of the global score and score by dimensions of the QOL answers of individuals infected with HIV and attended by the NGO | NGO  Answers to the quality of life questionnaire | 12 months |

| **Table 8.9** | | | |
| --- | --- | --- | --- |
| **PROVIDE FLATS FOR HIV+ INDIVIDUALS REGARDLESS OF GENDER** | | | |
| **Promote shelter and rehabilitation of seropositive individuals without economic resources** | | | |
| **PROCESS MEASURE** | **Indicator description** | **Method of Data collection** | **Data Collection Frequency** |
| Evaluation of vulnerability when providing flats for HIV+ individuals | Evaluation of vulnerability when providing flats for HIV+ individuals | NGO | 12 months |
| Monitoring of the number of spots in flats for HIV+ individuals in vulnerable situations | Number of spots in flats for HIV+ individuals in vulnerable situations | NGO | 12 months |
| Monitoring the number of HIV+ individuals in vulnerable situations that live in flats | Number of HIV+ individuals in vulnerable situations that live in the flats | NGO | 12 months |
| Monitoring of the number occupational training courses, new technology training courses, and social and employment insertion training courses for occupants of flats | Number of occupational training courses, new technology training courses, and social and employment insertion training courses for occupants of flats | NGO | 12 months |
| Monitoring of the number of monthly outsides to promote personal development | Number of monthly outsides to promote personal development  (theatre, museums, sports activities...etc. | NGO | 12 months |
| Monitoring of the duration of each type of outing | Total duration of each type of outing | NGO | 12 months |
| Monitoring of progress evaluation of the social and employment insertion of occupants of flats for HIV+ individuals | Progress evaluation of the social and employment insertion of occupants of flats for HIV+ individuals | NGO  In depth personal interviews | 12 months |
| **OUTCOME MEASURE** | **Indicator description** | **Method of Data collection** | **Data Collection Frequency** |
| Descriptive analysis and monitoring of QOL answers of individuals attended by the NGO | Descriptive analysis and monitoring of the global score and score by dimensions of the QOL answers of individuals infected with HIV and attended by the NGO | NGO  Anonymous user service questionnaire | 12 months |

| **Table 8.10** | | | |
| --- | --- | --- | --- |
| **TELEPHONE SERVICE, WEBSITE CONSULTATIONS, CHATS FOR SEROPOSITIVE INDIVIDUALS AND FOR SERODISCORDANT PARTNERS, FORUMS AND E-MAIL CONSULTATIONS** | | | |
| **Increase information for HIV+ individuals, via telephone, on-site and through websites** | | | |
| **PROCESS MEASURE** | **Indicator description** | **Method of Data collection** | **Data Collection Frequency** |
| Monitoring the total number of hours dedicated to personalized service via telephone | Total number of hours dedicated to personalized service via telephone | NGO | 12 months |
| Monitoring the total number of hours dedicated to personalized service via e-mail and Internet | Total number of hours dedicated to personalized service via e-mail and Internet | NGO | 12 months |
| Monitoring the number of individuals that hav received personalized service via telephone | Number of individuals that hav received personalized service via telephone / Number of phone calls received | NGO | 12 months |
| Monitoring of the number of individuals that have received personalized care via e-mail and Internet | Number of the number of individuals that have received personalized care via e-mail and Internet / Number of times the web office has been visited | NGO | 12 months |
| Monitoring of the number of individuals that have consulted the forum | Number of individuals that have consulted the forum / Number of times the forum has been visited | NGO | 12 months |
| **OUTCOME MEASURE** | **Indicator description** | **Method of Data collection** | **Data Collection Frequency** |
| Level of satisfaction with the activity | Number of individuals that have received personalized care through on-site, telephone, e-mail and Internet service, have responded to the satisfaction questionnaire and have a high level of satisfaction / Number of individuals that have received personalized care through on-site, telephone, e-mail and Internet service | NGO  Anonymous user service questionnaire | 12 months |
